# Berberine in combination with cisplatin induces necroptosis and apoptosis in ovarian cancer cells

**DOI:** 10.1186/s40659-019-0243-6

**Published:** 2019-07-18

**Authors:** Li Liu, Jingyan Fan, Guihai Ai, Jie Liu, Ning Luo, Caixia Li, Zhongping Cheng

**Affiliations:** 1 Department of Obstetrics and Gynecology, Shanghai Tenth People’s Hospital, Tongji University, 301 Yanchang Road, Shanghai, 200072 China; 2 Department of Obstetrics and Gynecology, Shanghai Ninth People’s Hospital, Jiaotong University, Shanghai, China

**Keywords:** Ovarian cancer, Berberine, Cisplatin, Apoptosis, Necroptosis

## Abstract

**Background:**

Berberine (BBR), a compound extracted from a variety of medicinal herbs, has been shown multiple pharmacological effects against cancer cells of different origins. Cisplatin (DDP) is known as an effective chemotherapeutic agent against cancer by inducing DNA damage and cell apoptosis. However, the effect of the combined used of BBR and DDP on cell necroptosis in ovarian cancer has not been reported.

**Methods:**

OVCAR3 and three patient-derived primary ovarian cancer cell lines (POCCLs) were chosen as the experimental objects. To determine the potential anti-cancer activity of BBR and DDP in combination, we firstly treated OVCAR3 and POCCLs cells with BBR and/or DDP. The cell viability of OVCAR3 and POCCLs with treatment of BBR or DDP for different hours was measured by CCK-8 assay. Flow cytometry was used to analyze cell cycle distribution and changes in apoptotic cells after treatment with BBR and/or DDP. The morphological changes of OVCAR3 cells were observed by using Transmission electron microscopy (TEM) analysis. Proliferation, apoptosis and necroptosis related markers of OVCAR3 and POCCLs with treatment of BBR or DDP were measured by RT-qPCR, western blotting and immunofluorescence assay.

**Results:**

Our results demonstrated that BBR significantly inhibited the proliferation of OVCAR3 and primary ovarian cancer cells in a dose- and time-dependent manner. The combination treatment of BBR and DDP had a prominent inhibitory effect on cancer cell growth and induced G0/G1 cell cycle arrest. TEM revealed that the majority of cells after BBR or DDP treatment had an increasing tendency of typical apoptotic and necrotic cell death morphology. Besides, BBR and DDP inhibited the expression of PCNA and Ki67 and enhanced the expression and activation of Caspase-3, Caspase-8, RIPK3 and MLKL.

**Conclusion:**

This study proposed that the combination therapy of BBR and DDP markedly enhanced more ovarian cancer cell death by inducing apoptosis and necroptosis, which may improve the anticancer effect of chemotherapy drugs. The apoptosis involved the caspase-dependent pathway, while the necroptosis involved the activation of the RIPK3–MLKL pathway. We hope our findings might provide a new insight for the potential of BBR as a therapeutic agent in the treatment of ovarian cancer.

## Background

Ovarian cancer (OC) remains the most lethal form of all gynecologic malignancies worldwide. More than two-thirds of patients are diagnosed at an advanced stage due to insidious onset, leading to poor prognosis [1, 2]. Cytoreductive surgery followed by chemotherapy is the current standard of care for patients.

Cisplatin (DDP) and its derivatives are commonly used agents for OC chemotherapy. The therapeutic mechanism of these DNA-damaging agents, particularly DDP, seems to be associated with their efficacy in inhibiting the proliferation and apoptosis of cancer cells [3–5]. However, serious side effects after administration of DDP was found in patients, including bone marrow suppression, nephrotoxicity, neurotoxicity and gastrointestinal reactions. To tackle this problem, researchers pinned their hope on combination therapy. Several researches have been done in striving to find drugs find drugs that enhance the anticancer effect of DDP without increasing side effects, but failed.

Berberine (BBR), an isoquinoline derivative alkaloid isolated from Huang Lian and other Chinese medicinal herbs, has been extensively used for the treatment of various clinical diseases such as diabetes, metabolic syndrome and diarrhea [6–8]. Recent evidence suggests that BBR possesses anticancer activities involving multiple biological mechanisms for a wide variety of cancers, including liver cancer, glioblastoma, breast cancer and OC [9–12]. BBR exhibits an inhibitory effect on cell proliferation and induces apoptosis in OC cells. In addition, BBR has been demonstrated to induce cell apoptosis through directly binding with DNA and interfering with DNA replication as DNA topoisomerase I inhibitor. Another study showed that BBR sensitized OC cells to DDP through miR-93/PTEN/Akt signaling pathway, and BBR enhanced DDP induced apoptosis [12–14].

Programmed cell death, such as apoptosis, necroptosis and autophagy, plays crucial roles in the physiopathological processes of the organism, as well as in the pathogenesis and progress of cancer [15, 16]. Previous studies were mainly focused on the mechanism of apoptosis in cancer, and little attention has been paid to the roles of necroptosis and autophagy in the pathogenesis and progress of cancer [17, 18]. However, whether BBR exerts the anticancer effect by inducing tumor cell necroptosis remains unanswered. The aim of the present study was to investigate the effect of BBR and DDP on human ovarian cell line OVCAR3 and three patient-derived primary OC cell lines (POCCLs) by simultaneous observation of the ability of either BBR or DDP alone or both in inducing apoptosis and necroptosis in these cells, hoping that our study could provide new insights into the anticancer mechanism of BBR and DDP.

## Materials and methods

### Reagents

Reagents used in this study were BBR and DDP (Sigma, St. Louis, MO, USA); antibodies for Ki67, PCNA, Caspase3, Clv C3, Caspase8, Clv C8, RIPK3, p-RIPK3 and p-MLKL (Abcam, Cambridge, MA, USA); antibodies for Cyclin D1 and glyceraldehyde-3-phosphate dehydrogenase (GAPDH) (Cell Signaling Technologies, Danvers, MA, USA); antibody for MLKL (Novus Biologicals, Littleton, CO, USA); and secondary antibodies of donkey anti-goat HRP, goat anti-rabbit HRP and goat anti-mouse HRP (Beyotime Institute of Technology, China).

### Ethics statement

All three patients enrolled in this study underwent surgery of primary OC at Yangpu Hospital affiliated to Tongji University School of Medicine (Shanghai, China). The study was approved by the Research Ethics Committee of Tongji University, and in accordance with the tenets and guidelines of the Declaration of Helsinki. Written informed consent was obtained from all patients.

### Cell collection and culture

Human OC OVCAR3 cells were obtained from the Chinese Type Culture Collection of the Chinese Academy of Sciences (Shanghai, China). All three patient-derived POCCLs were established after sterile processing of the samples from surgical biopsies obtained at diagnostic radical surgeries of OC patients who were diagnosed as malignant Fesddration Internationale des Gynaecologistes et Obstetristes (FIGO) stage III serous adenocarcinomas. The detailed procedures for the isolation of these cell lines were as previously described [19–21]. OVCAR3 cells and POCCLs were cultured in DMEM (hyclone) and D/F12 (hyclone) respectively, supplemented with 10% fetal bovine serum (FBS, Gibco), 100 U/mL penicillin sodium, and 100 mg/mL streptomycin sulfate (Solarbio) at 37 °C in a humidified air atmosphere containing 5% CO_2_. Cells were used when they were in the logarithmic growth phase.

### Cell proliferation assay

Cells (5.0 × 10^3^/well) were plated, treated in 96-well plates, and stimulated with different concentrations of BBR (50, 100, 200, 500 μM) or/and DDP (5 mg/L) for 24, 48 and 72 h [22]. Cell proliferation was detected by using the Cell Count Kit-8 (CCK-8, Signalway Antibody) according to the manufacturer’s instructions. Absorbance was detected at a wavelength of 450 nm with a microplate reader (Bio-Rad). Half-maximal inhibitory concentration (IC50) of OVCAR3 cells after BBR treatment 24 h was calculated from an experimentally derived dose–response curve for each concentration by using GraphPad Prism 6.0 software for Windows (GraphPad Software, La Jolla, CA, USA) [23]. Each assay was carried out in triplicate.

### Cell cycle analysis

The cell cycle was evaluated by flow cytometry using PI (7 Seabiotech, Shanghai, China) staining on a flow cytometer (BD Biosciences). Cells were plated in 6-well plates, treated with BBR (100 μM) or/and DDP (5 mg/L) for 24 h, washed in PBS, and re-suspended in staining solution containing 20 μg/mL PI and 100 μg/mL RNase A. Experiments were performed in triplicate and 3 × 10^4^ cells were analyzed per sample. G1, S, and G2/M fractions were quantified with the FlowJo software (Tree Star). The experiments were repeated in triplicate.

### Annexin V-FITC/PI apoptosis assay

Apoptotic cells were analyzed using an Annexin V-FITC/PI double staining procedure. Cells were treated with BBR (100 μM) or/and DDP (5 mg/L) for 24 h. Indicated cells were digested into single cell suspensions using EDTA-free trypsin and then stained according to the instructions provided with the Annexin V-FITC/PI Apoptosis Detection kit (Beyotime, Shanghai, China). The stained cells were analyzed in 10–15 min by flow cytometry (BD Biosciences). The apoptosis of OC cells was detected by determining the relative amount of Annexin V-FITC positive alone cells (early apoptosis) and both Annexin V-FITC and PI positive cells (late apoptosis) as previously described [24]. Both Annexin V-FITC and PI negative cells were considered as living cells. The group without any treatment was used as a blank control group. The relative apoptosis level of other groups was compared with the corresponding group as described in figure legend 4. At least 2 × 10^4^ cells were acquired for each sample. The experiments were performed in triplicate.

### Real-time polymerase chain reaction

Quantitative real-time PCR (qRT-PCR) was performed using the ABI 7300 instrument (Applied Biosystems, Foster City, CA) and SYBR Green chemistry (Thermo Fisher Scientific, Rockford, IL). The primers used were list in Table 1.

**Table 1:** List of designed primers for real-time polymerase chain reaction

Primer name	Primer sequence
*Caspase3* (NM_004346.3)	Forward: 5′ GTTTGAGCCTGAGCAGAGAC 3′ Reverse: 5′ TGGCAGCATCATCCACAC 3′
*Caspase*-*8*	Forward: 5′ TGTGCCCAAATCAACAAGAG 3′ Reverse: 5′ TTCAAAGGTCGTGGTCAAAG 3′
*Ki67*	Forward: 5′ GTGCGAAGGTTCTCATGC 3′ Reverse: 5′ CTTGACACTCCGCGTTAC 3′
*PCNA*	Forward: 5′ GCTCTTCCCTTACGCAAGTC 3′ Reverse: 5′ AGTGCCTCCAACACCTTC 3′
*RIPK3*	Forward: 5′ CGCCTGCTGAAAGAAGTG 3′ Reverse: 5′ GTGAGCCTCCCTGAAATG 3′
*MLKL*	Forward: 5′ CCTGTTTCACCCATAAGC 3′ Reverse: 5′ GACTGCCTCAAAGTTTCC 3′
*GAPDH*	Forward: 5′ CACCCACTCCTCCACCTTTG 3′ Reverse: 5′ CCACCACCCTGTTGCTGTAG 3′

*GAPDH* was used as a normalizing gene. OC cells (5.0 × 10^5^/well) were plated and treated in 6-well plates after 24 h with BBR (100 μM) or/and DDP (5 mg/L). Total RNA was extracted using TRIzol Reagent (invitrogen) according to the manufacturer’s instructions. Complementary DNA was synthesized by reverse transcriptase at 37 °C for 1 h and 85 °C for 5 min. The PCR cycling conditions were as follows: 95 °C for 7 min, followed by 40 cycles of 15 s at 95 °C and 60 °C for 45 s. Verification of specific product amplification was determined by dissociation curve analysis. The gene expression was calculated using the∆∆CT method [25]. All data represent the mean of three replicates.

### Western blot analysis

In order to balance the decrease in the number of cells caused by the agents, we collected 2 × 10^6^ cells per group for Western blot protein extraction. Cell lysates were prepared with radio-immunoprecipitation assay (RIPA) buffer containing protease and phosphatase inhibitors. The protein concentration was measured by bicinchoninic acid assay (BCA, Thermo Fisher Scientific). The supernatant with an equal amount of protein was separated on SDS-PAGE gels. Proteins then were blotted onto nitrocellulose membranes and incubated with primary antibodies and the corresponding secondary antibodies. The membranes were developed with enhanced chemiluminescence (BioRad, Richmond, CA). GAPDH served as an internal control. Western blot bands were measured with the ImageJ software (National Institutes of Health, USA) to analyze the integrated density value (IDV). The average IDV values of indicated proteins with GAPDH were compared and the average relative value was obtained. Then we normalized the average relative value of control group to 1, and the relative protein level of other groups was obtained by comparison with the control group. Each assay was carried out in triplicate.

### Transmission electron microscopy analysis

Cells were fixed in 2% glutaraldehyde for 2 h and washed two times with PBS for 10 min. The cells were then fixed in 1% OsO4 for 2 h. After gradient dehydration with ethanol, the cells were embedded in epoxy resin and cut into 50–60 nm sections. The sections were stained with uranyl acetate combined with lead citrate and observed under a Philips QUANTA-200 transmission electron microscope.

### Immunofluorescence assay

1 × 10^5^ OVCAR3 cells were plated in 12-well chamber slides and treated with or without agents for 24 h. The cells were fixed with 4% paraformaldehyde at room temperature for 30 min and washed 3 times with 0.02 M phosphate buffered saline (PBS) at room temperature for 3 min and incubated with blocking solution (PBS, 3% of BSA, 0.5% Triton-X 100) at room temperature for 3 min. Antibodies against PCNA, Ki67, Clv C8, Clv C3, RIPK3 and MLKL in primary antibody diluent (PBS, 3% BSA, 0.5% Triton-X 100) was added and incubated at 4 °C overnight; cells were washed with PBS, incubated with secondary antibody at room temperature for 1 h (goat anti-rabbit the Alexa Fluor 555 and goat anti-mouse FITC), and washed 3 times with PBS at room temperature for 10 min. Images were obtained with a fluorescence microscope (DP70; Olympus).

### Statistical analysis

All data analysis was performed using SPSS 20.0 software for Windows. Results were shown as the mean ± SD or mean ± SEM. Comparisons between groups were made by one-way analysis of variance (ANOVA) followed by Student–Newmann–Keuls test. p-values < 0.05 were considered statistically significant and each experiment was repeated in triplicate.

## Results

### BBR inhibits cell proliferation and enhances the inhibitory effect of DDP in OC cells

**Fig. 1:**
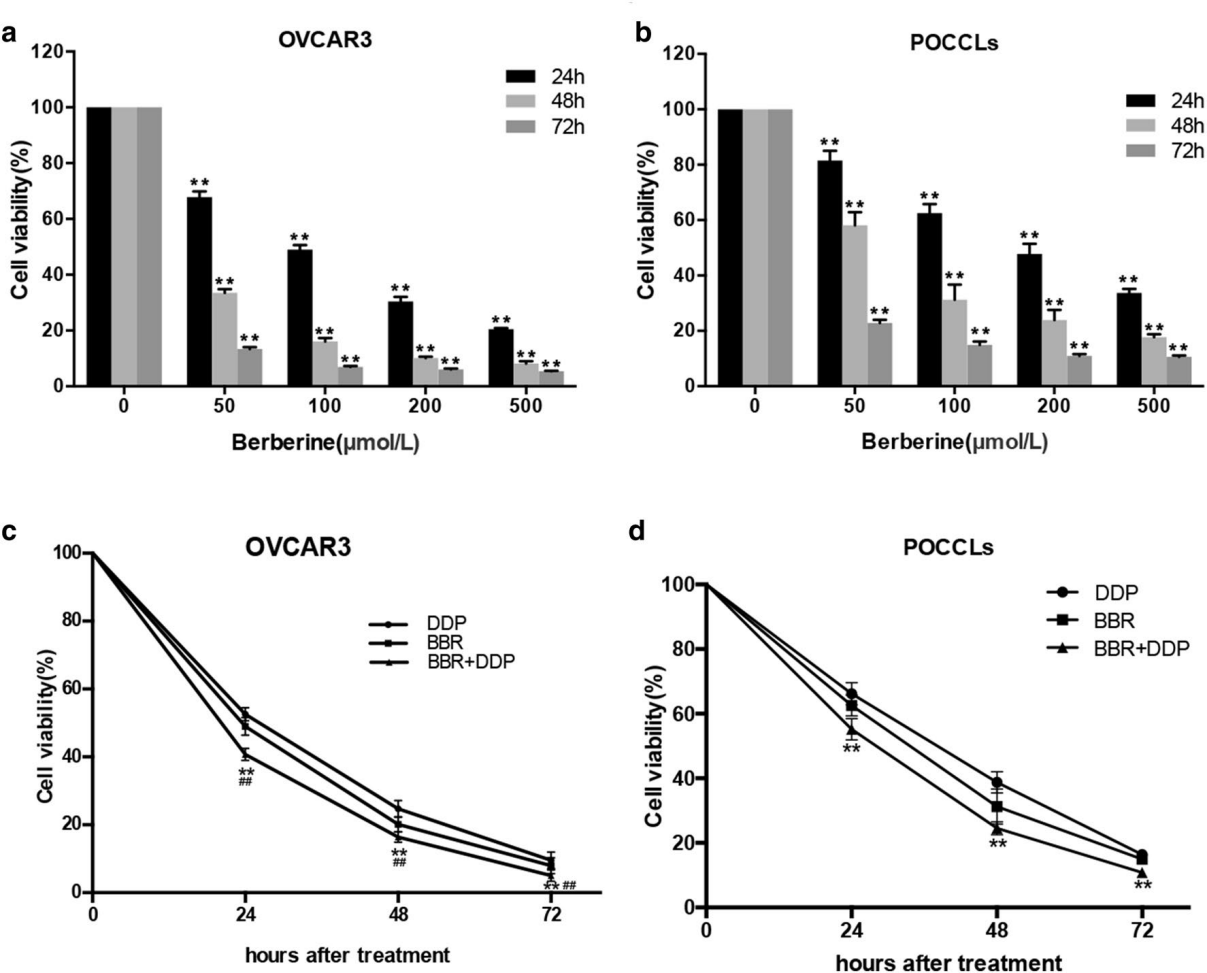
BBR inhibited cell proliferation and enhanced the inhibitory effect of DDP when they were used in combination. OVCAR3 cells (**a**) and POCCLs (**b**) were exposed to various concentrations of BBR for the indicated periods. *p < 0.05, **p < 0.01 compared with the respective 24 h, 48 h and 72 h controls. The inhibitory effect of BBR (100 μM) in combination with DDP (5 mg/L) on OVCAR cells (**c**) and POCCLs (**d**) after 24-h treatment. Cell viability was measured by CCK-8 assay. *p < 0.05, **p < 0.01 compared with DDP group. ^#^p < 0.05, ^##^p < 0.01 compared with BBR group. Data were based on at least three independent experiments, and shown as mean ± SD

**Fig. 2:**
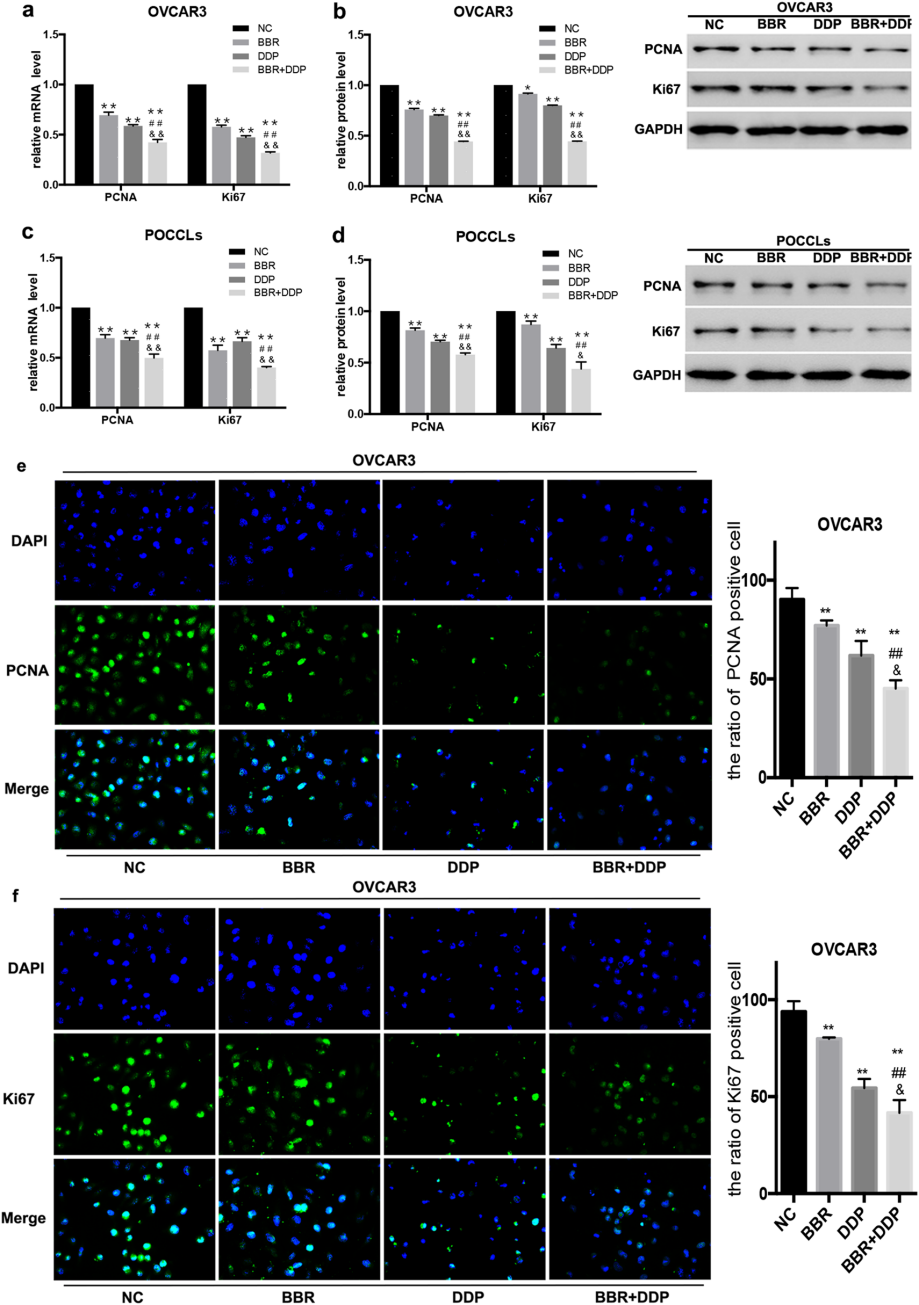
BBR + DDP combination inhibited the expression of PCNA and Ki67. OVCAR3 cells and POCCLs were treated with BBR (100 μM) and/or DDP (5 mg/L) for 24 h. The expression level of Ki67 and PCNA in BBR and/or DDP-treated cells was detected by qRT-PCR (**a**, **c**), Western blot (**b**, **d**) and immunofluorescence assay (**e**, **f**). Data were based on at least three independent experiments, and shown as mean ± SEM. *p < 0.05, **p < 0.01 compared with NC; ^#^p < 0.05, ^##^p < 0.01 compared with BBR; ^&^p < 0.05, ^&&^p < 0.01 compared with DDP

To determine the potential anti-cancer activity of BBR and DDP in combination, we firstly treated OVCAR3 cells and POCCLs cells with increasing concentrations of BBR for different hours (24–72 h). Cell viabilities were measured and quantified by CCK-8 assay. The results obtained show that BBR significantly inhibited cell viability in OVCAR3 (Fig. 1a) and POCCLs (Fig. 1b) in a dose- and time-dependent manner compared to corresponding blank control group (NC), with the greatest effect at 72 h at a concentration of 500 μmol/L BBR in both cases. The 50% inhibitory concentration (IC_50_) value of OVCAR3 cells was 99 ± 1.58 μM at 24 h after BBR treatment, and therefore the concentration of 100 μM was chosen as the optimal BBR concentration in the following experiments. According to the published literature, The IC_50_ value of DDP on OVCAR3 at 24 h was 13.23 ± 2.83 μM [26]. In order to investigate the combination anti-cancer effects of BBR and DDP, we selected DDP 5 mg/L, a cytotoxic dose close to its IC50, as appropriate concentration of DDP in this study. Our data showed that the inhibitory effect of co-treatment of BBR and DDP was significantly higher than that of either BBR or DDP alone (Fig. 1c, d). Meanwhile, the expression level of proliferating cell nuclear antigen (PCNA) and Ki67 involved in cell proliferation was evaluated by real-time PCR and Western blot in each groups. As shown in Fig. 2a–d, mRNA and protein levels of proliferation markers PCNA and Ki67 were significantly suppressed in OVCAR3 cells and POCCLs after treatment with either BBR or DDP, and this effect was enhanced in both cell lines co-treated with the two compounds comparing to that of either agent treatment alone. Similar results were also observed in immunofluorescence assay (Fig. 2 e, f).

### BBR combined with DDP induces G1-phase cell cycle arrest in OC cells

**Fig. 3:**
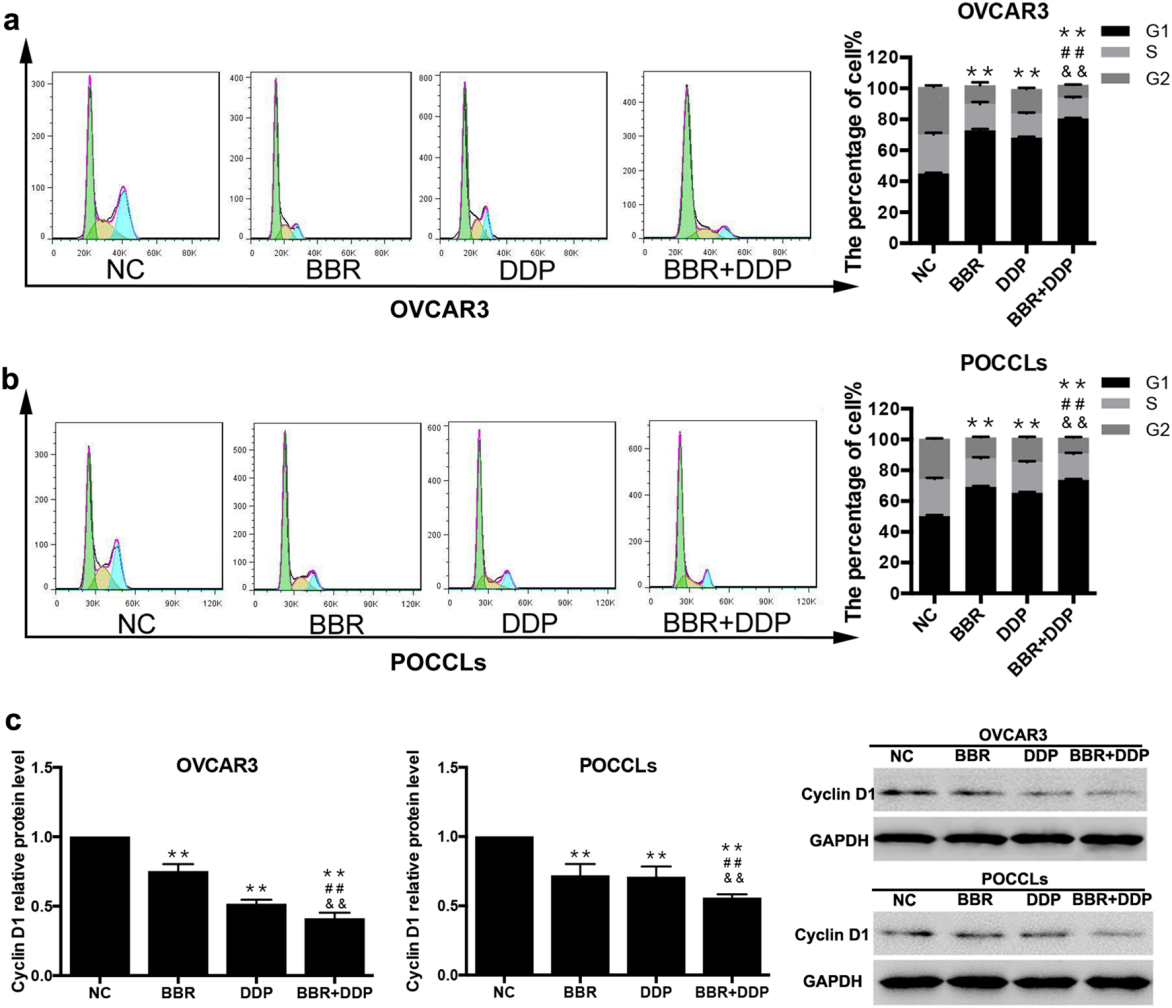
BBR + DDP induced G1-phase cell cycle arrest. OVCAR3 cells (**a**) and POCCLs (**b**) were treated with BBR (100 μM) and/or DDP (5 mg/L) for 24 h. The cell cycle was evaluated by flow cytometry using PI staining. The expression level of Cyclin D1 in BBR and/or DDP-treated cells was detected by qRT-PCR and Western blot (**c**). Data were based on at least three independent experiments, and shown as mean ± SEM. *p < 0.05, **p < 0.01 compared with NC; ^#^p < 0.05, ^##^p < 0.01 compared with BBR; ^&^p < 0.05, ^&&^p < 0.01 compared with DDP

To determine whether BBR and DDP affected cell cycle of OC cells, cell cycle distribution was assessed by flow cytometry. Our results showed that BBR or DDP significantly increased the number of both OVCAR3 cells (Fig. 3a) and POCCLs (Fig. 3b) in G0/G1 phase as compared with control group, and this effect was even enhanced in co-treatment groups of BBR and DDP as compared with the groups of single treatment. Supporting these results, the expression levels of cell cycle regulation protein, Cyclin D1, which is involved in promoting G1/S transition were evaluated by real-time PCR and western blot. The mRNA and protein levels of Cyclin D1 were decreased after either BBR or DDP treated alone, while combination treatment of them caused less Cyclin D1 expression both in OVCAR3 cells and POCCLs (Fig. 3c).

### Co-treatment of BBR and DDP induces apoptosis in OC cells through extrinsic caspase-dependent pathway

**Fig. 4:**
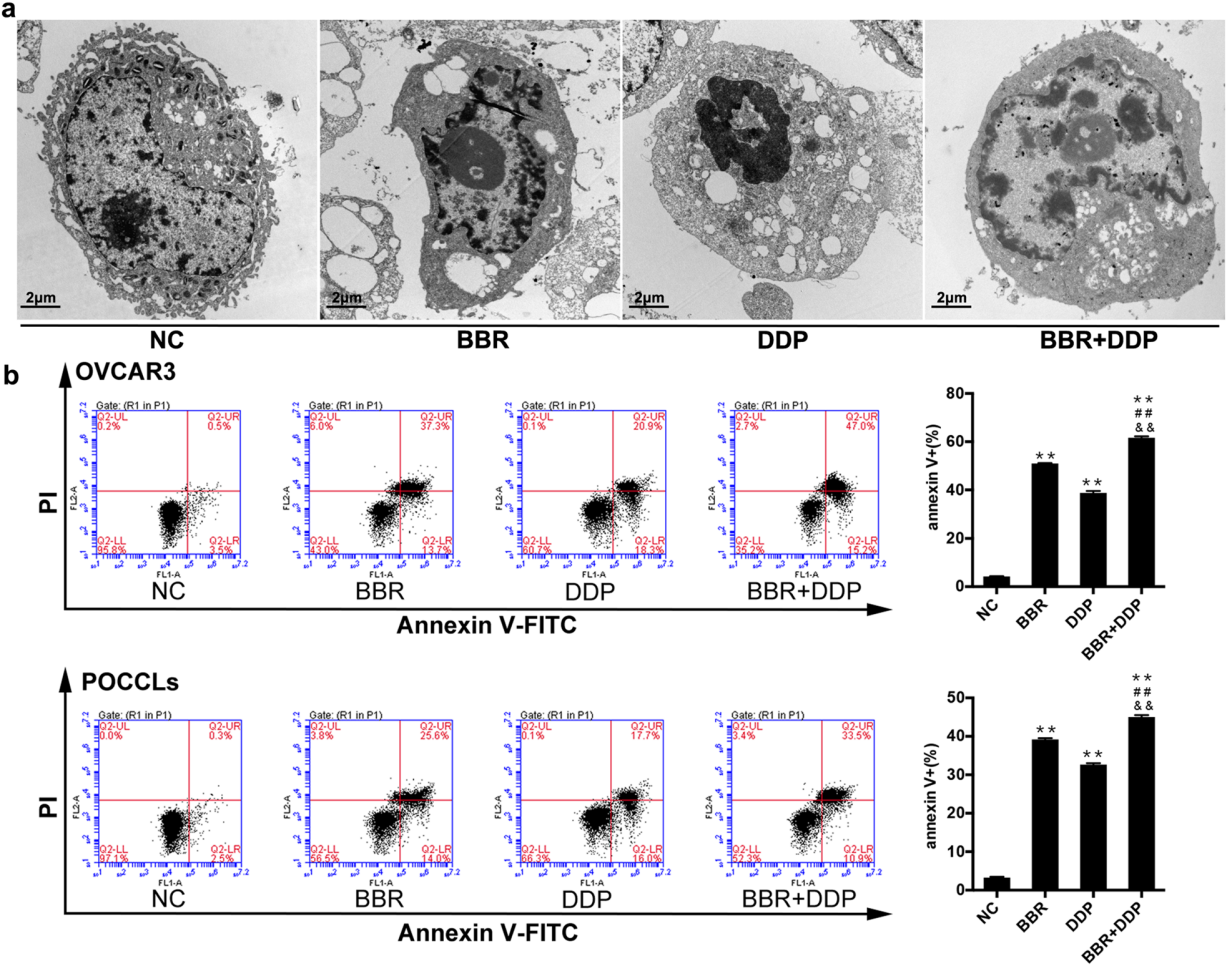
BBR + DDP combination induced apoptosis. OVCAR3 cells (**a**) and POCCLs (**b**) were treated with BBR (100 μM) and/or DDP (5 mg/L) for 24 h. The gross changes and apoptotic morphology of OVCAR3 cells were detected by Transmission Electron Microscopy (TEM) assay under ×4200 magnification (**a**). The number of OVCAR3 and POCCLs undergoing apoptosis was detected by flow cytometry using Annexin V-FITC/PI double staining (**b**). Data were based on at least three independent experiments, and shown as mean ± SEM. *p < 0.05, **p < 0.01 compared with NC; ^#^p < 0.05, ^##^p < 0.01 compared with BBR; ^&^p < 0.05, ^&&^p < 0.01 compared with DDP

To explore the apoptotic function of BBR and DDP-treated OC cells, we first observed the morphological changes of OVCAR3 cells by using TEM. As shown in Fig. 4a, TEM revealed that BBR or DDP induced a significant number of OVCAR3 cells with apoptosis changes mainly include cytoplasmic shrinkage, blebbing of the plasma membrane, chromatin condensation and the formation of apoptotic bodies, while combination treatment of BBR and DDP induced more apoptotic cells. Next, our results from Annexin V-FITC/propidium iodide (PI) staining indicated that BBR or DDP significantly induced cell apoptosis in both OVCAR3 cells and POCCLs compared with corresponding control cells. The apoptotic rates induced by BBR + DDP combination was significantly higher than that by either BBR or DDP alone (Fig. 4b).

**Fig. 5:**
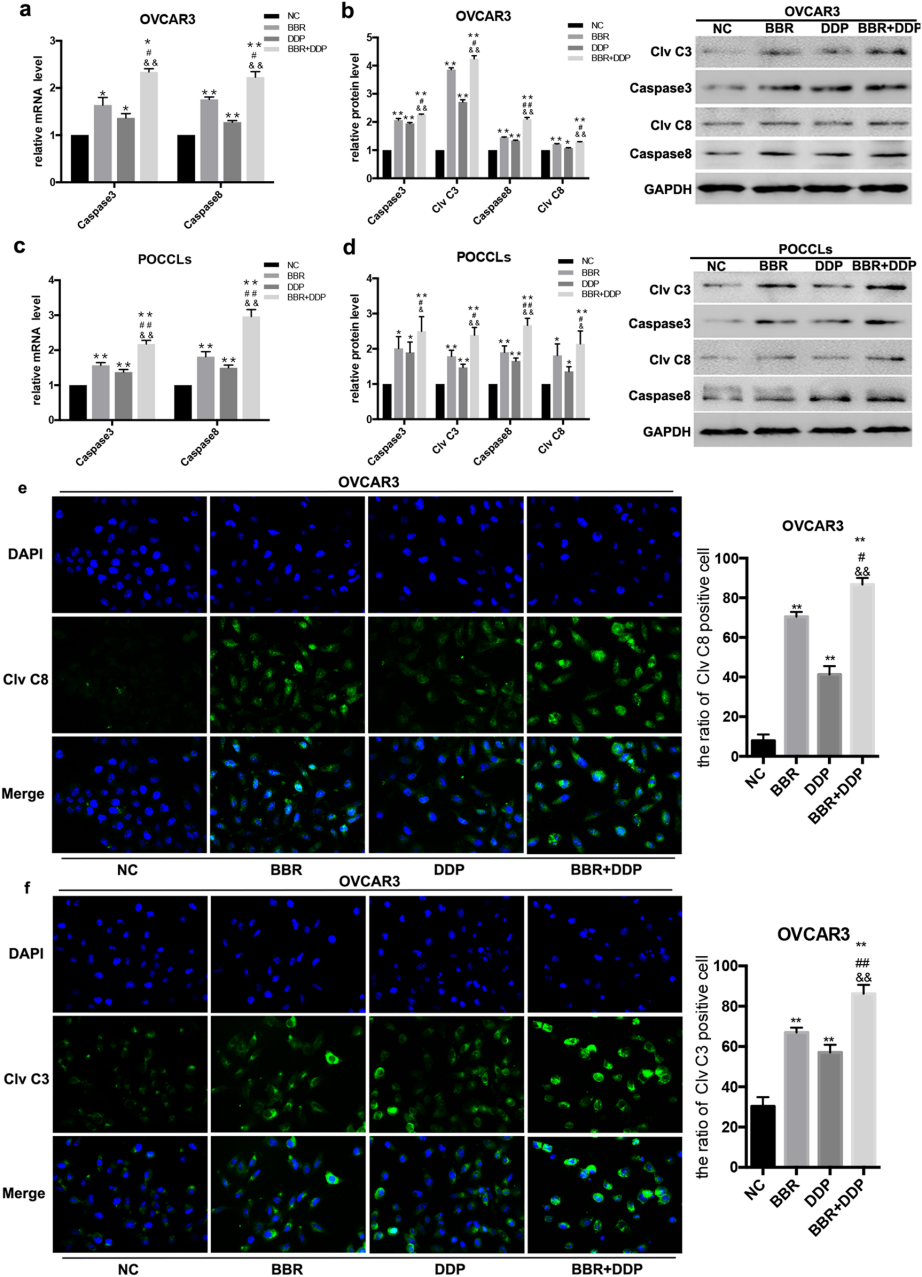
BBR + DDP combination enhanced Caspase3 and Caspase8 activities. OVCAR3 cells and POCCLs were treated with BBR (100 μM) and/or DDP (5 mg/L) for 24 h. The expression levels of Caspase3, Caspase8, cleaved Caspase3 and cleaved Caspase8 in BBR and/or DDP-treated cells was detected by qRT-PCR (**a**, **c**) and Western blot (**b**, **d**). Immunofluorescence assay (**e**, **f**) also showed that the expression levels and subcellular localization of cleaved Caspase8 (Clv C8) and cleaved Caspase3 (Clv C3) in OVCAR3 cells. Data were based on at least three independent experiments, and shown as mean ± SEM. *p < 0.05, **p < 0.01 compared with NC; ^#^p < 0.05, ^##^p < 0.01 compared with BBR; ^&^p < 0.05, ^&&^p < 0.01 compared with DDP

Interestingly, our results showed that the mRNA (Fig. 5a, b) and protein (Fig. 5c, d) levels of Caspase8 and Caspase3 were significantly increased in both OVCAR3 cells and POCCLs treated with BBR or DDP alone as compared with corresponding control cells. Meanwhile, the protein level of the cleavages of both Caspase8 (Clv C8) and Caspase3 (Clv C3) were also significantly increased in both OVCAR3 cells and POCCLs treated with BBR or DDP alone as compared with corresponding control cells by using immunoblotting assay (Fig. 5c, d). Similar results were also observed in immunofluorescence assay of OVCAR3 cells (Fig. 5e, f) The combination treatment of the BBR and DDP caused more expression and activation of Caspase8 and Caspase3 in both OVCAR3 cells and POCCLs comparing to single treatment groups of BBR or DDP. Collectively, these results suggested that co-treatment of BBR and DDP induced apoptotic cell death through Caspase8–Caspase3 pathway in OC cells.

### BBR and DDP induces necroptosis in OC cells through RIPK3–MLKL pathway

**Fig. 6:**
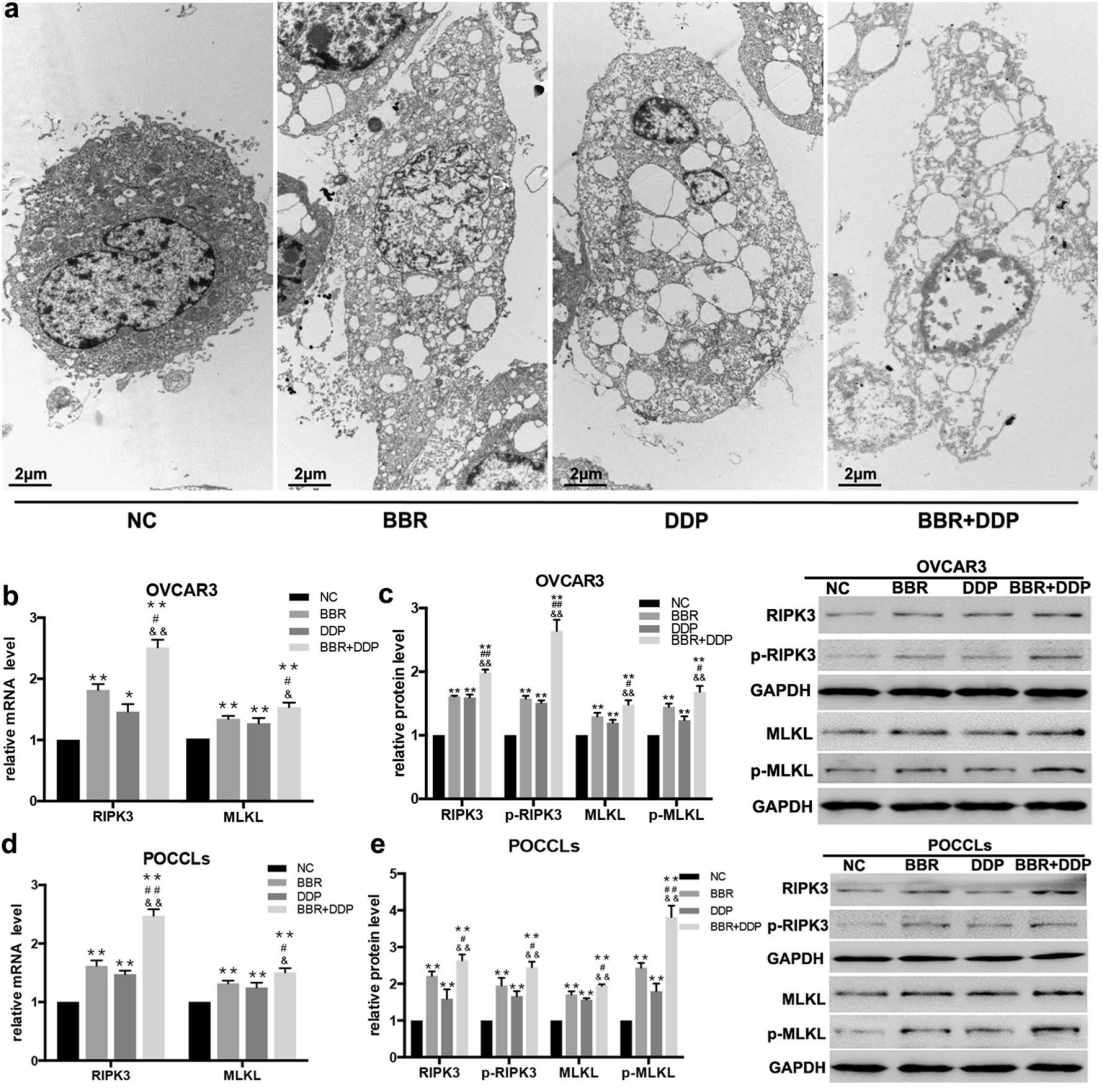
BBR and DDP enhanced the expression and activation of RIPK3 and MLKL. OVCAR3 cells and POCCLs were treated with BBR (100 μM) and/or DDP (5 mg/L) for 24 h. The gross changes and necrotic morphology of OVCAR3 cells were detected by transmission electron microscopy (TEM) assay under ×4200 magnification (**a**). The expression levels of RIPK3 and MLKL in BBR and/or DDP-treated cells were detected by qRT-PCR (**a**, **b**) and Western blot (**c**, **d**). The phosphorylation of RIPK3 and MLKL was also detected by immunoblotting (**c**, **d**). Data were based on at least three independent experiments, and shown as mean ± SEM. *p < 0.05, **p < 0.01 compared with NC; ^#^p < 0.05, ^##^p < 0.01 compared with BBR; ^&^p < 0.05, ^&&^p < 0.01 compared with DDP

**Fig. 7:**
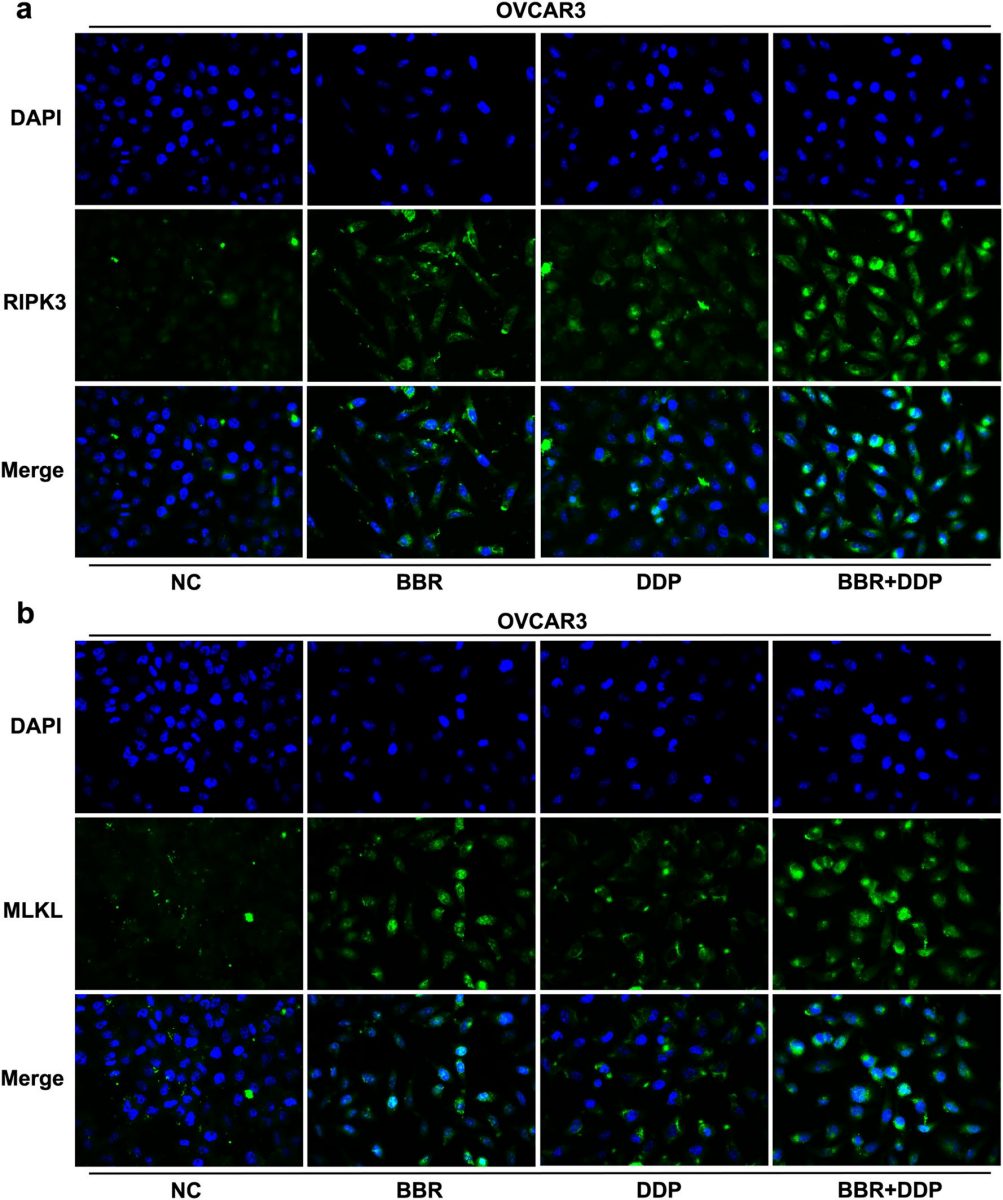
Immunofluorescence showed the expression of RIPK3 and MLKL in OVCAR3 cells. OVCAR3 cells were treated with BBR (100 μM) and/or DDP (5 mg/L) for 24 h. The expression levels and subcellular localization of RIPK3 (**a**) and MLKL (**b**) were detected by immunofluorescence assay. Data were based on at least three independent experiments

To see whether BBR and DDP affected necroptosis of OC cells, we also observed the morphological changes of OVCAR3 cells by using TEM analysis. As shown in Fig. 4a, TEM revealed that majority of cells after BBR or DDP treatment had a typical necrotic cell death morphology as manifested by the extensive vesiculation of cytoplasmic organelles and rupture of the plasma membrane, while combination treatment of BBR and DDP induced more necrotic cells. Besides, the expression and activation of receptor-interacting serine/threonine-protein kinase 3 (RIPK3) and mixed lineage kinase domain-like (MLKL) as the biomarkers of necroptosis were detected by real-time PCR and Western blot. The result showed that either BBR or DDP alone significantly enhanced the mRNA and protein levels of RIPK3 and MLKL in OVCAR3 cells (Fig. 6a, c) and POCCLs (Fig. 6b, d) as compared with the control group. The increasing levels of these biomarkers and its subcellular localization pattern in OVCAR3 cells were also observed using immunofluorescent assay (Fig. 7a, b). The phosphorylation of RIPK3 (p-RIPK3) and MLKL (p-MLKL) was increased in either BBR- or DDP-treated group as shown by immunoblotting. Similar results were also obtained in BBR + DDP combination group as compared with either BBR or DDP alone.

## Discussion

In the present study, we evaluated the combined effects of BBR and DDP on OVCAR3 and 3 patient-derived primary ovarian cancer cell lines. Our data demonstrated that combination treatment of BBR and DDP markedly enhanced the anti-cancer effects of DDP by inducing apoptosis and necroptosis in ovarian cancer. The apoptosis involved the caspase-dependent pathway, while the necroptosis involved the activation of the RIPK3–MLKL pathway. Taken together, BBR combined with DDP markedly inhibited cell growth and might provide a new therapeutic strategy for the clinical treatment of ovarian cancer.

Platinum-based chemotherapy remains the main treatment of patients with advanced or recurrent OC. However, three-decade clinical observations seem to suggest that the overall survival rate of OC has not been improved significantly. Clinically, a huge number of patients relapse after receiving first-line chemotherapy, and the side-effects of chemotherapy is serious, which is associated with the poor prognosis [27, 28]. Therefore, we focus on the combination of DDP and other novel agents from natural products with low toxicity. Traditional Chinese medicines have aroused increasing attention and interest in the field of chemotherapy for OC recently [29–31]. Studies have shown that BBR as an isoquinoline derivative alkaloid has shown an antiproliferative effect against a variety of human cancer cells and is relatively safe for normal cells. Sudheer reported that treatment of normal human prostate epithelial cells with BBR (10–100 μM) did not significant affect their viability in vitro [32]. Another study showed that no significant reproductive toxicity was observed in pregnant rats treated with berberine 500–600 times more than human dosage [33]. As for cancer, Huang et al. [9] reported that BBR sensitized human liver cancer cells to sorafenib via inhibiting the proliferation of cancer cells and markedly induced cellular apoptosis. In basal-like breast cancer, BBR bound to vasodilator-stimulated phosphoprotein to induce the change of its secondary structure, and inhibited breast cancer cell proliferation and migration finally [34]. Besides, by binging to retinoid X receptor alpha to suppress β-catenin signaling, BBR eventually inhibited the growth of colon cancer cells [35]. Our current study confirmed such findings. Besides, in comparison with BBR or DDP treatment alone, it was found that the combination of BBR and DDP had a prominent inhibitory effect on OVCAR3 and POCCLs growth. Afore-mentioned experimental results were also supported by the significant decrease in mRNA and protein expressions of the proliferative markers PCNA, Ki67. However, it must be pointed out that the dose of DDP we choose came from published literature, and previous data showed the IC_50_ value of DDP on OVCAR3 was 13.23 ± 2.83 μM [26]. In order to detect the growth-inhibitory effect of DDP in ovarian cancer cell lines, Zhang et al. [26] detected IC50 of DDP on six different ovarian cancer cell lines at 24, 48, 72 h respectively and their data showed the IC50 value of DDP on OVCAR3 at 24 h was 13.23 ± 2.83 μM. Herein, we selected 5 mg/L (i.e. 16.67 μM) of DDP a cytotoxic dose close to its IC_50_ in this study to investigate the combination anti-cancer effects with BBR, and our CCK-8 result on OVCAR3 (Fig. 1c) showed that our concentration selection was relatively consistent with previous study.

Cell cycle disorder is an important mechanism of tumorigenesis, and most chemotherapeutic drugs act on this target. Our study also confirmed BBR and DDP arrested OVCAR3 cells and POCCLs proliferation at the G0/G1 phase, which was accompanied by down-regulating the protein levels of Cyclin D1. Similar studies have been reported. In breast cancer, cells were arrested by BBR at the G1 phase via increasing the expression of p21/cip1 [36]. BBR-mediated G0/G1 phase cell cycle arrest was also observed in Huh-7 and HepG2 cells [37]. Another report indicated that BBR inhibited DNA topoisomerase I via binging with DNA [13].

Apoptosis, an autonomous cell death, involves the activation, expression, and regulation of various proteins, which is strictly controlled by genes. Clinically, inducing cellular apoptosis is an important mechanism of chemotherapeutic drugs to control tumor development [15, 16]. It has been reported that BBR can induce apoptosis in various cancer cells. As an example, in HeLa cells, BBR combined with DDP enhanced apoptosis through a mitochondria/caspase-mediated pathway [38]. Besides, BBR enhanced DDP-induced apoptosis through the miR-21/PDCD4 axis [39]. We also confirmed that BBR combined with DDP significantly induced cell apoptosis in both OVCAR3 cells and POCCLs through Annexin V-FITC/propidium iodide (PI) staining.

Necroptosis, a controllable cell death patterns, has proved to be wildly involved in the physiopathological processes of the organism, and plays an important role in the pathogenesis and progress of cancers [40–42]. Chemotherapeutic drugs induced necroptosis has been reported in tumor. It has been found that DDP induced necroptosis through the TNFα-mediated RIPK1/RIPK3/MLKL pathway. On the other hand, DDP opened mitochondrial permeability transition pore to produce reactive oxygen species, which eventually led to necroptosis [43]. Another study also showed that some natural compounds such as shikonin, neoalbaconol, and tea polyphenols stimulated necroptosis of cancer cells [44]. However, there is no report indicating the combined effects with BBR and DDP on necroptosis in OC. Therefore, this study was designed to simultaneously observe the effects of BBR and DDP on both apoptosis and necroptosis in OC. Generally, apoptosis and necroptosis can be induced via the cognate ligands of the death receptor family, such as tumor necrosis factor receptor 1 (TNFR1). The activation of Caspase-8 is considered to play a central role in the mechanism of TNF-mediated apoptosis. Its classical function consists of cleaving and activating downstream caspases, such as Caspase-3 and Caspase-7, and pro-apoptotic proteins to promote mitochondrial damage and apoptosis [45]. Necroptosis can be activated upon stimulation by ligands of TNFα when cells are deficient for Caspase-8 or its adaptor protein FADD, or in the presence of a caspase inhibitor. The activation of RIPK3 is a critical upstream event in necroptosis. MLKL, a functional RIPK3 substrate, is phosphorylated by at Ser358 leads to its oligomerization and the interaction of phosphorylated MLKL with phosphatidylinositol phosphates (PIPs) allows the recruitment of MLKL to the plasma membrane, where it forms pores to promote cell lysis, allowing necroptosis to occur [40–42, 45–47]. In the present study, we found that both pathways were activated. Transmission electron microscopy provides a direct visual way, we observed a large number of OVCAR3 cells with typical morphological changes of apoptosis and necrosis. Furthermore, we combined BBR and DDP to stimulate OC cells to evaluate the therapeutic effect of the combination. The result showed that the combination of 100 μM BBR and 5 mg/L DDP inhibited cell proliferation and induced apoptosis and necroptosis even more strongly, as evidenced by the increased apoptotic and necrotic numbers, higher expression and activation of Caspase3, Caspase8, RIPK3 and MLKL, when compared with either BBR or DDP treated alone. Taken together, these data suggest that BBR may enhance the anti-cancer effect of DDP by inhibiting proliferation and inducing apoptosis and necroptosis of ovarian cancer cells.

## Conclusions

In summary, our study has demonstrated that the combination of BBR and DDP can inhibit proliferation and induce both apoptosis and necroptosis of OC cells. This finding may shed light on the potential application of BBR to the clinical treatment of cancers, and provide a new therapeutic strategy for the clinical treatment of OC when it is combined with DDP in the future. We consider it necessary to evaluate the different death types of cancer cells simultaneously for the sake of gaining a more comprehensive understanding of the biologic effects of various anti-cancer agents. However, the molecular mechanism associated with BBR/DDP-mediated apoptosis and necroptosis in OC remains poorly understood, and further investigation in vivo and prospective clinical cancer trials are needed for the clinical application of BBR.

## Data Availability

The datasets used and/or analyzed during the current study are available from the corresponding author on reasonable request.

## Abbreviations

BBR: berberine
DDP: cisplatin
OC: ovarian cancer
POCCLs: primary OC cell lines
TEM: transmission electron microscopy
GAPDH: glyceraldehyde-3-phosphate dehydrogenase
qRT-PCR: quantitative real-time PCR
RIPA: radio-immunoprecipitation assay
IDV: integrated density value
PBS: phosphate buffered saline
NC: blank control group
PCNA: proliferating cell nuclear antigen
Clv C8: the cleavages of Caspase8
Clv C3: the cleavages of Caspase3
RIPK3: receptor-interacting serine/threonine-protein kinase 3
MLKL: mixed lineage kinase domain-like
TNFR1: tumor necrosis factor receptor 1
PIPs: phosphatidylinositol phosphates
